# Varied Mulching Practices Modulate Soil Hydrothermal-Nutrient Regimes and Root Yield of Indigowoad Root in an Oasis

**DOI:** 10.3390/plants15203161

**Published:** 2026-10-15

**Authors:** Peng Zhou, Zeyi Wang, Yucai Wang, Fuqiang Li, Xuan Li, Caixia Huang, Lian Lei

**Affiliations:** 1 College of Water Conservancy and Hydropower Engineering, Gansu Agricultural University, Lanzhou 730070, China; zhoupeng200205@163.com (P.Z.);; 2 Gansu Provincial Agricultural Smart Water-Saving Technology Innovation Center, Lanzhou 730070, China; 3 Yimin Irrigation Experimental Station, Minle 734500, China

**Keywords:** Indigowoad Root, mulching practices, soil hydrothermal conditions, soil nutrients, yield, PLS-PM

## Abstract

Water scarcity and plastic film pollution increasingly threaten the sustainable production of Indigowoad Root (*Isatis tinctoria* L.) in semi-arid regions. However, the mechanisms by which different mulching practices under flat planting regulate soil hydrothermal conditions, nutrient availability, and yield remain poorly understood. The study aimed to provide theoretical and technical support for water-saving and high-yield production of Indigowoad Root in oasis areas. Bare soil without mulching was used as the control (CK), and five mulching treatments were established: biodegradable white plastic film mulching (DW), biodegradable black plastic film mulching (DB), white polyethylene film mulching (PW), black polyethylene film mulching (PB), and straw mulching (SM). The effects of these treatments on soil water storage, soil temperature, soil organic matter, soil nutrient status, leaf area index, dry matter accumulation, and yield of Indigowoad Root were evaluated. The mulching treatments improved soil hydrothermal conditions and nutrient status and promoted plant growth and yield formation in Indigowoad Root. Compared with CK, DB significantly increased soil water storage and soil temperature. It also increased the contents of ammonium nitrogen, nitrate nitrogen, available phosphorus, and available potassium, thereby promoting dry matter accumulation and yield formation. The PLS-PM results indicated that improved soil hydrothermal conditions enhanced soil nutrient availability, thereby promoting plant growth and driving yield formation (increased by 25%). Biodegradable black film mulching (DB) increased Indigowoad Root yield by improving soil hydrothermal conditions, enhancing soil nutrient availability, and promoting plant growth, thereby providing a promising water-saving and yield-enhancing mulching strategy for Indigowoad Root production in the semi-arid Hexi Oasis.

## 1. Introduction

Indigowoad Root (*Isatis Tinctoria* L.) grown in the Hexi region is a medicinal cash crop unique to the cool and arid areas of northwestern China, its planting area has remained above 6667 ha for many years, with an annual production of approximately 30,000 t [1]. It contains active constituents such as indigo, indirubin, and (R,S)-goitrin. Both its roots and leaves are used medicinally and are widely used in the clinical practice of traditional Chinese medicine and the manufacture of related preparations [2]. However, annual precipitation is low and unevenly distributed among seasons in the main production areas, resulting in severe water stress during the growth of Indigowoad Root [3,4]. As a biennial herbaceous medicinal plant, Indigowoad Root has a root system that extends vertically into deep soil layers, with most roots concentrated within the 0–60 cm soil profile [2]. This distinctive root architecture makes the plant highly sensitive to soil conditions and dependent on continuous and stable supplies of water and nutrients. Consequently, soil water and nutrient dynamics directly affect dry matter accumulation and quality formation in Indigowoad Root. Conventional tillage and management practices cause substantial evaporative water loss from soil and severe nutrient loss, creating a marked mismatch between the soil supply of water and nutrients and crop growth requirements. This mismatch ultimately results in pronounced interannual variation in yield and inconsistent quality. Optimizing cultivation practices to improve the soil environment and increase water and nutrient use efficiency is therefore important for the stable production of high-quality Indigowoad Root and the sustainable development of the Indigowoad Root industry.

Mulching is an important agronomic practice for regulating the soil microenvironment in dryland agriculture. It provides substantial benefits by improving soil hydrothermal conditions and soil nutrient status and modulating the structure of soil microbial communities [5,6]. Previous studies have shown that organic mulch materials, such as maize as well as wheat straw, add organic matter to the soil through decomposition [7,8]. Such organic mulch materials can significantly increase plant height, ear height, and water use efficiency in maize [9,10], improve canopy photosynthetic performance and nutrient accumulation in wheat [11,12], and enhance tuber quality and marketable tuber percentage in potato [13,14]. They can also improve the soil microenvironment for fruit trees and promote their growth and development [15,16].

Plastic film mulching suppresses weeds and provides moderate soil-warming and moisture-conservation effects, thereby creating a suitable rhizosphere environment for crop growth [17,18]. Plastic mulch films are generally made of polymeric materials, among which polyethylene (PE) film is widely used because of its low cost, stable performance, and effective soil-warming and moisture-conservation properties. However, its long-term use and incomplete recovery can lead to the accumulation of residual film and microplastics in soil [19,20]. Biodegradable mulch films can gradually decompose through microbial activity under suitable conditions and are regarded as promising alternatives to conventional PE films, although their degradation performance is affected by material composition, soil properties, and climatic conditions [20]. Mulch-film color also alters the absorption, reflection, and transmission of solar radiation, thereby regulating soil hydrothermal conditions, weed growth, and soil microbial communities and ultimately affecting crop growth and yield formation [21,22].

The yield-enhancing and water-saving effects of different mulching practices have been extensively studied in crops such as maize, wheat, potato, and fruit trees [9,10,11,12,13,14,15,16]. However, deep-rooted, taproot biennial crops differ fundamentally from other crops in how they acquire and use soil water and nutrients and respond to soil thermal conditions [23]. Nevertheless, the effects of different mulching practices on the soil environment and yield of Indigowoad Root remain poorly studied and understood. In particular, few studies have conducted multi-year comparisons of biodegradable films, conventional polyethylene films, films of different colors, and straw mulch under identical field conditions. Moreover, limited attention has been paid to their integrated effects on soil water storage, soil temperature, nutrient status, plant growth, and final yield. To address this knowledge gap, we conducted a field experiment using bare soil without mulch as the control and established five mulching treatments: biodegradable white plastic film, biodegradable black plastic film, white polyethylene film, black polyethylene film, and straw mulch. The study aimed to systematically evaluate the effects of these mulching practices on soil water storage, soil temperature, soil organic matter content, nutrient status, leaf area index, dry matter accumulation, and yield of Indigowoad Root in a semi-arid region. We hypothesized that: (1) all mulching treatments would reduce soil water loss to some extent, whereas their effects on soil temperature would vary according to mulch material and film color; (2) biodegradable films would provide soil water conservation and yield benefits comparable to those of conventional polyethylene films during the growing season, although different film colors would induce distinct soil hydrothermal conditions and plant responses; and (3) mulching would affect leaf area development, dry matter accumulation, and final yield of Indigowoad Root primarily by regulating soil moisture, temperature, and nutrient availability. The study was intended to provide a theoretical and technical basis for developing a green cultivation system that conserves soil moisture, suppresses evaporation, reduces reliance on conventional polyethylene film, and improves efficiency in Indigowoad Root production in the semi-arid regions of northwestern China.

## 2. Materials and Methods

### 2.1. Experimental Site

**Figure 1:**
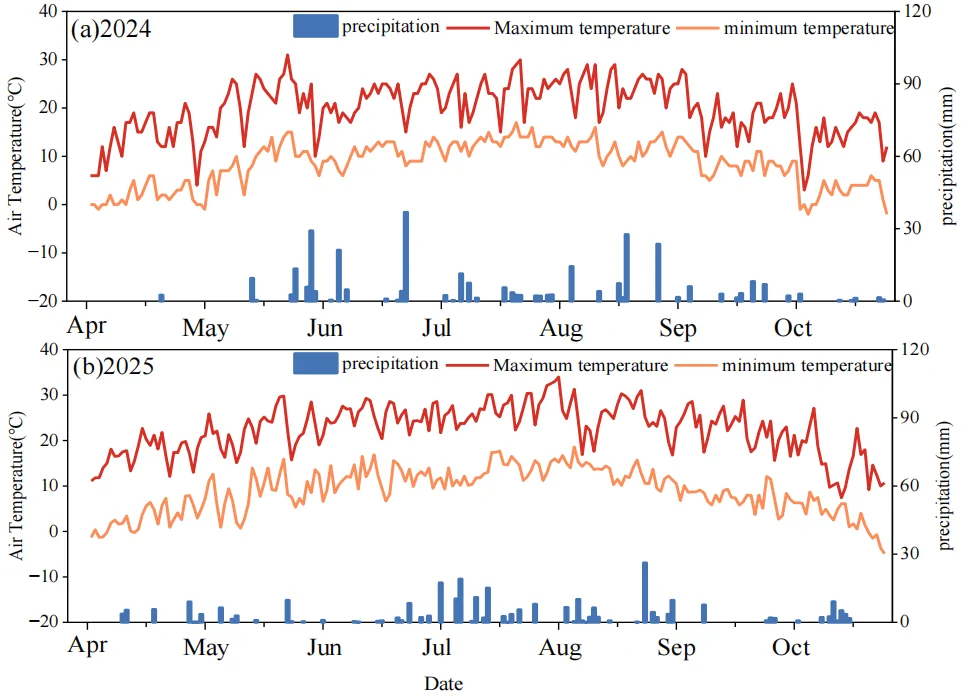
Precipitation and air temperature during the growing seasons of Indigowoad Root at the experimental site in 2024 and 2025.

The field experiment was conducted in 2024 and 2025 at the Yimin Irrigation Experimental Station in Minle County, Gansu Province, China (38°39′ N, 100°43′ E; elevation, 1970 m). The experimental area has a temperate continental desert-steppe climate, with a mean annual air temperature of 6.0 °C and an annual sunshine duration of approximately 3000 h. Mean annual precipitation ranges from 183 to 285 mm, whereas annual evaporation is 1015 mm. The extreme minimum and maximum air temperatures are −33.3 and 37.8 °C, respectively, and the frost-free period ranges from 109 to 174 d. Daily precipitation and daily maximum and minimum air temperatures during the experimental period are presented in Figure 1.

The soil texture at the experimental site was light loam. Before initiation of the field experiment, the baseline fertility of the 0–20 cm plow layer was determined. Soil bulk density was 1.40 g cm^−3^, and the contents of ammonium nitrogen, nitrate nitrogen, available phosphorus, and available potassium were 64.59, 87.90, 43.83, and 128.54 mg kg^−1^, respectively.

### 2.2. Experimental Design

**Figure 2:**
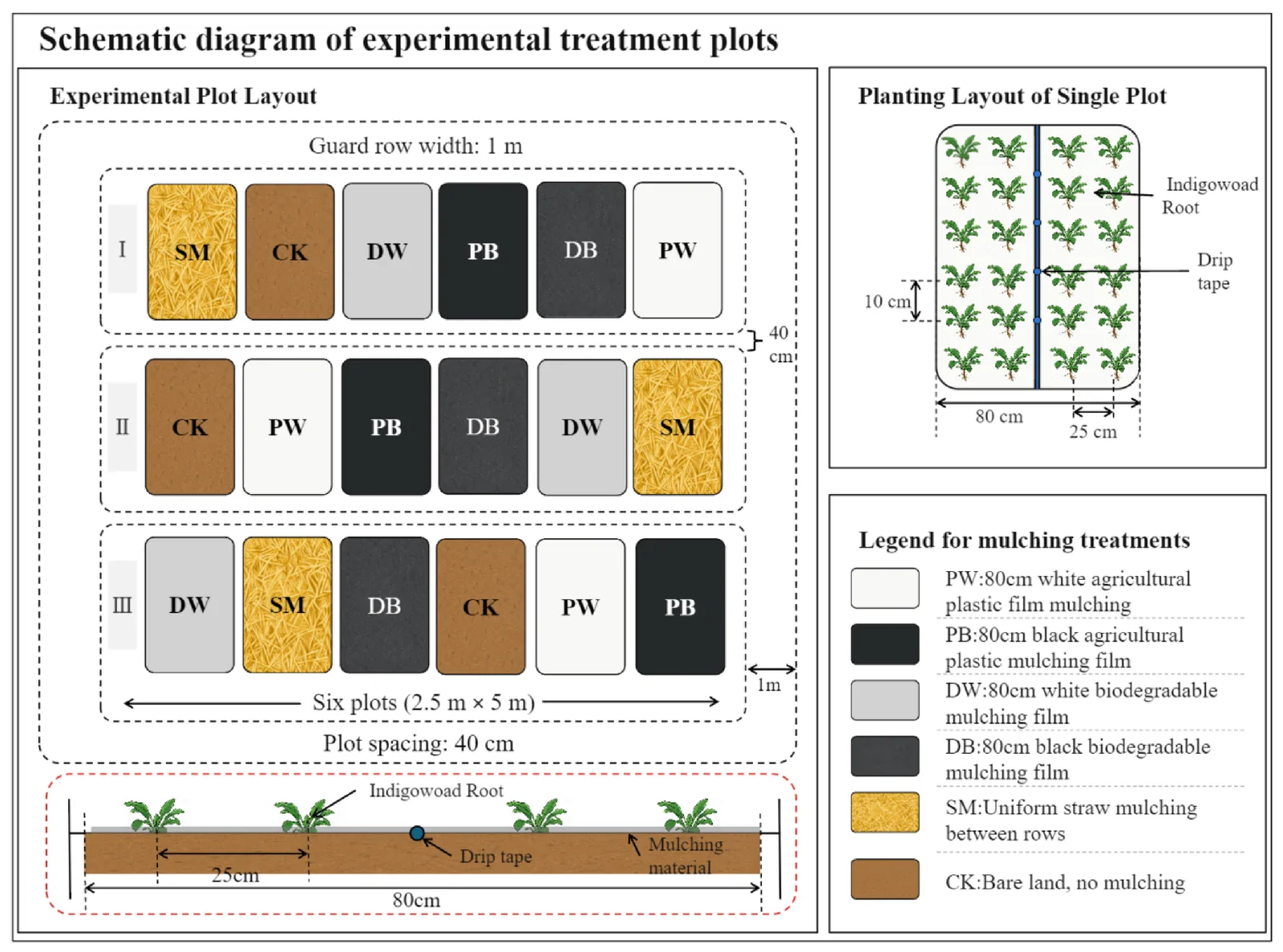
Schematic illustration of the experimental design, planting configuration, and mulching treatments for Indigowoad Root. Roman numerals I–III indicate the three replicate blocks. CK, bare soil without mulching (control); PW, white polyethylene film mulching; PB, black polyethylene film mulching; DW, biodegradable white plastic film mulching; DB, biodegradable black plastic film mulching; and SM, straw mulching.

The experiment was arranged in a randomized block design. Flat planting with bare soil and no mulch was used as the control (CK), and five mulching treatments under flat planting were established: white polyethylene film mulching (PW), black polyethylene film mulching (PB), biodegradable white plastic film mulching (DW), biodegradable black plastic film mulching (DB), and straw mulching (SM)(Xinjiang Tianye Group Co., Ltd., Shihezi, China). Each treatment was replicated three times, and each plot had an area of 12.5 m^2^ (2.5 m × 5.0 m). The field layout is shown in Figure 2.

In 2024, Indigowoad Root was sown on 30 April and harvested on 12 October. In 2025, it was sown on 2 May and harvested on 15 October. The within-row plant spacing and row spacing were 10 and 25 cm, respectively. Three days before sowing, basal fertilizer was incorporated into the soil at a depth of 25 cm using an integrated tillage and fertilization machine. The application rates of nitrogen (N), phosphorus (P), and potassium (K) were 225, 90, and 150 kg ha^−1^, The nitrogen, phosphorus, and potassium fertilizers used in the experiment were urea (N ≥ 46%,Xinxinlian Chemical Group Co., Ltd., Xinxiang, China), superphosphate (P_2_O_5_ ≥ 52%,Yunnan Yuntianhua Co., Ltd., Kunming, China), and potassium sulfate (K_2_O ≥ 52%,SDIC Lop Nur Sink Potash Co., Ltd., Korla, China), respectively. One day before sowing, the DB, DW, PB, and PW films were laid, and new films were used before sowing each year. For SM, maize straw was spread evenly over the soil surface at a rate of 4500 kg ha^−1^. The irrigation amount followed the local conventional irrigation quota of 202 m^3^ ha^−1^. Irrigation was applied three times, at the seedling, rosette, and fleshy-root enlargement stages, respectively. All other field management practices followed local practices.

### 2.3. Measurements and Methods

#### 2.3.1. Soil Water Content and Soil Water Storage

Soil samples were manually collected with a soil auger from the 0–20, 20–40, and 40–60 cm soil layers. Sampling was conducted at the seedling, rosette, fleshy-root expansion, and harvest stages. Soil water content was determined using the oven-drying gravimetric method by drying the soil samples at 105 °C to constant mass.

Soil water content was calculated as follows [24]:

(1) $$\theta_{i}=\frac{M_{\mathrm{fi}}-M_{\mathrm{di}}}{M_{\mathrm{di}}}$$

where $\theta_{i}$ is the soil water content of the (i)th soil layer (%), $M_{\mathrm{fi}}$ is the wet soil mass of the (i)th layer (g), $M_{\mathrm{di}}$ is the oven-dry soil mass of the (i)th layer (g), and (i) is the soil-layer number.

Soil water storage was calculated as follows:

(2) $$\mathrm{SWS}=\sum_{i=1}^{n}(\theta_{i}\times Z_{i}\times \rho_{i}/100)$$

where SWS is soil water storage (mm), Z_i_ is the thickness of the (i)th soil layer (mm), and $\rho_{i}$ is soil bulk density (g cm^−3^).

#### 2.3.2. Soil Temperature

Soil temperature data loggers (HY-1, Yongkang, China) were buried in each treatment plot at depths of 5, 10, 15, 20, and 25 cm. Soil temperature was recorded at 2 h intervals at each growth stage of Indigowoad Root, and mean values were calculated.

#### 2.3.3. Determination of Soil Nutrients

At each growth stage of Indigowoad Root, soil samples were collected from the 0–20, 20–40, and 40–60 cm soil layers. The collected samples were homogenized and passed through a 1 mm sieve for the determination of soil physicochemical properties.

Soil organic matter was determined by potassium dichromate oxidation with external heating [25]. Soil ammonium nitrogen was determined by indophenol blue colorimetry [26]. Soil nitrate nitrogen was extracted with potassium chloride and determined by ultraviolet spectrophotometry [27]. Soil available phosphorus was determined by molybdenum–antimony–ascorbic acid colorimetry [28]. Soil available potassium was extracted with 1 mol L-1 ammonium acetate and determined using a flame photometer [29].

#### 2.3.4. Determination of Dry Matter Accumulation

At each growth stage, three representative Indigowoad Root plants were sampled from each plot. Each plant was separated into leaves and roots. The samples were placed in a drying oven at 105 °C for 0.5 h to halt enzymatic activity and were then transferred to an oven at 80 °C and dried to constant mass. The dry masses of the leaves and roots were recorded separately and used to quantify dry matter accumulation in Indigowoad Root.

#### 2.3.5. Determination of Leaf Area Index

At each growth stage, three uniformly growing Indigowoad Root plants were selected from the middle row of each plot. For each measured variable, the mean of the three sample plants was used as the observation for that plot, which represented one replicate. Each treatment had three replicate plots.

The leaves of the sample plants were collected. The length and width of each leaf were measured, and individual leaf area was calculated as length × width × 0.75. The green leaf area of each plant was calculated by summing the individual areas of all its leaves. Leaf area index (LAI) was defined as the ratio of green leaf area to the corresponding ground area and was calculated using the following equation:

(3) LAI=LA×PD/10,000

where LA is the green leaf area per plant (m^2^ plant^−1^), PD is planting density (plants ha^−1^), and 10,000 is the land-area conversion factor (m^2^ ha^−1^).

#### 2.3.6. Yield Determination

At harvest, a 1 m^2^ quadrat was selected from the center of each plot for fresh root yield measurement. After soil adhering to the roots and other debris had been removed, the roots were weighed and the plants were counted to determine plant number and fresh root yield at the population level. Fresh root yield for each plot was estimated from the quadrat and expressed on a per-hectare basis using the following equation:

(4) $$Y=\left(M/A\right)\times 10^{4}$$

where Y is the fresh root yield of Indigowoad Root (kg ha^−1^), M is the fresh mass of roots within the quadrat (kg), and A is the harvested area of the quadrat (m^2^).

### 2.4. Data Analysis

Data were compiled using Microsoft Excel 2025(Microsoft Corporation, Redmond, WA, USA). One-way analysis of variance (ANOVA) was performed using IBM SPSS Statistics 26.0 (IBM, Armonk, NY, USA), and Duncan’s multiple range test was used for multiple comparisons. A partial least squares path model (PLS-PM) was constructed using SmartPLS 4.0 (SmartPLS GmbH, Oststeinbek, Germany). Figures were prepared using Origin 2025(OriginLab Corporation, Northampton, MA, USA).

## 3. Results

### 3.1. Effects of Different Mulching Practices on Soil Water Storage During Indigowoad Root Growth

**Figure 3:**
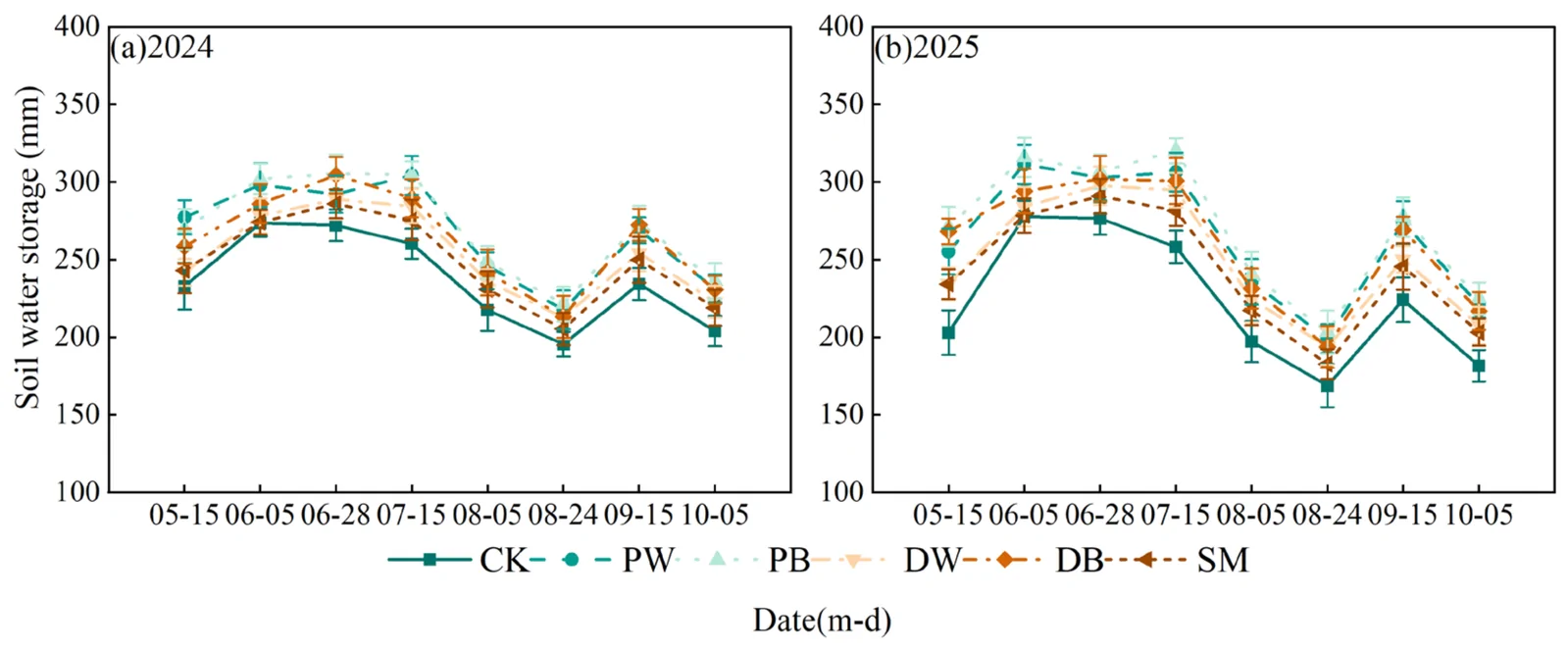
Temporal variation in soil water storage within the 0–60 cm soil profile under different mulching treatments during the growing seasons of Indigowoad Root. Values are presented as means ± standard errors (SE; n = 3), with vertical bars indicating the SE.

As shown in Figure 3, the soil water storage values and percentage increases reported for the different treatments represent averages across the two experimental years. Soil water storage generally increased initially and then decreased as plant growth progressed. The overall treatment ranking was DB > PW > PB > DW > SM > CK. In most cases, soil water storage was higher under all mulching treatments than under CK. At the seedling stage, soil water storage under DB, PW, and PB was 12%, 11%, and 5% higher than that under CK, respectively (*p* < 0.05), whereas DW and SM did not differ significantly from CK (*p* > 0.05). At the rosette stage, all five mulching treatments had greater soil water storage than CK. At the fleshy-root expansion stage, soil water storage gradually decreased, while the treatment ranking remained DB > PW > PB > DW > SM > CK. No significant differences were detected among the five mulching treatments; however, all had greater soil water storage than CK. At harvest, soil water storage decreased further. Compared with CK, soil water storage under DB, PW, PB, DW, and SM was 20%, 18%, 16%, 12%, and 10% higher, respectively. Soil water storage under DB, PW, and PB was significantly greater than that under CK, whereas DW and SM did not differ significantly from CK. Overall, DB generally maintained the highest soil water storage across the growth stages, while soil water storage under PW, PB, DW, and SM was also generally higher than that under CK.

### 3.2. Effects of Different Mulching Practices on Soil Temperature During Indigowoad Root Growth

**Figure 4:**
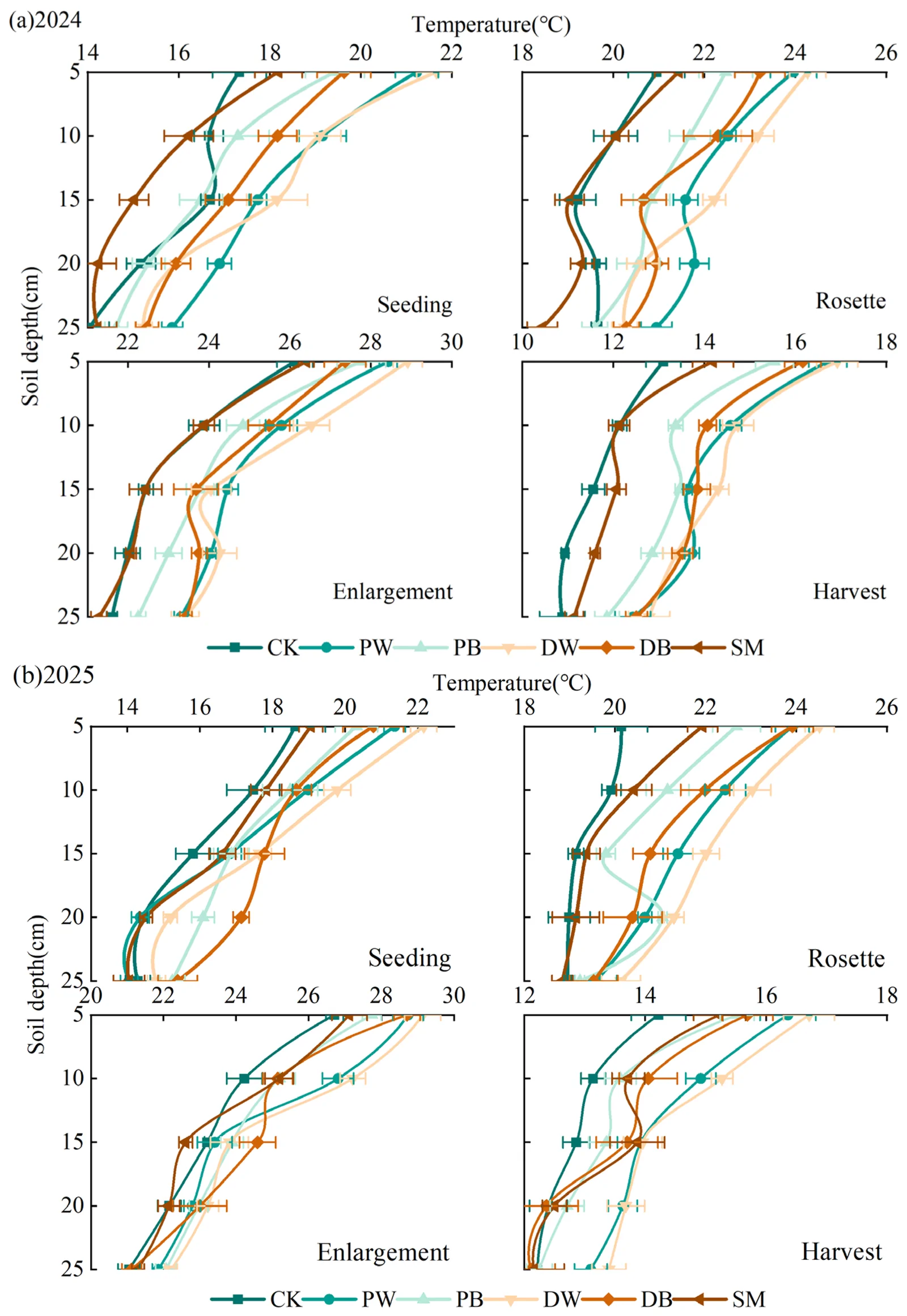
Soil temperature within the 0–25 cm soil layer at different growth stages of Indigowoad Root under different mulching treatments. Values are means ± SE (n = 3).

As shown in Figure 4, soil temperature initially increased and subsequently decreased over the course of crop development and generally decreased with increasing soil depth across treatments. At the seedling stage, soil temperature at the 5 cm depth ranged from 17.3 to 22.2 °C and followed the order DW > DB > PW > PB > SM > CK. Compared with CK, DW and DB significantly increased soil temperature by 22% and 12%, respectively (*p* < 0.05). At the 10 cm depth, soil temperature under DW and DB was significantly higher than that under CK by 2.4 and 1.4 °C, respectively (*p* < 0.05), whereas the temperatures under PW, PB, and SM were similar to that under CK. At the 15-, 20-, and 25 cm depths, DB produced the highest soil temperatures, exceeding those under CK by 1.2, 1.7, and 1.7 °C, respectively. At the rosette stage, the highest soil temperatures were recorded at the 5 cm depth. At this depth, DW and DB significantly increased soil temperature by 3.8 and 3.0 °C, respectively, compared with CK (*p* < 0.05), and the treatments followed the order DW > DB > PW > PB > SM > CK. No significant difference was detected between SM and CK (*p* > 0.05). At the 10 cm depth, DW and DB significantly increased soil temperature by 16% and 11%, respectively, relative to CK (*p* < 0.05), whereas PW, PB, and SM increased soil temperature by 2.5, 1.4, and 0.5 °C, respectively. At the 15 cm depth, soil temperatures under DW and PW were higher than those under the other treatments. At the 20- and 25 cm depths, PW produced the largest increases in soil temperature. At the fleshy-root enlargement stage, soil temperatures at the 5- and 10 cm depths followed the order DW > DB > PW > PB > SM > CK. Compared with CK, DW significantly increased soil temperature by 2.6 °C at the 5 cm depth and by 2.8 °C at the 10 cm depth (*p* < 0.05). At the 15-, 20-, and 25 cm depths, soil temperature was highest under DW, followed by DB, whereas PW, PB, and SM did not differ significantly from CK (*p* > 0.05). At harvest, soil temperature reached its lowest level over the entire growth period. At the 5 cm depth, soil temperature followed the order DW > PW > DB > PB > SM > CK, and DW significantly increased soil temperature by 3.2 °C compared with CK (*p* < 0.05). At the 10 cm depth, soil temperature under DW was significantly higher than that under CK by 2.4 °C (*p* < 0.05). Only small differences in soil temperature were observed among treatments at the 15-, 20-, and 25 cm depths. Overall, DW generally produced the strongest soil-warming effect, followed by DB, whereas soil temperature under SM was generally similar to that under the uncovered control (CK).

### 3.3. Effects of Different Mulching Practices on Soil Organic Matter and Nutrients

#### 3.3.1. Soil Organic Matter

**Figure 5:**
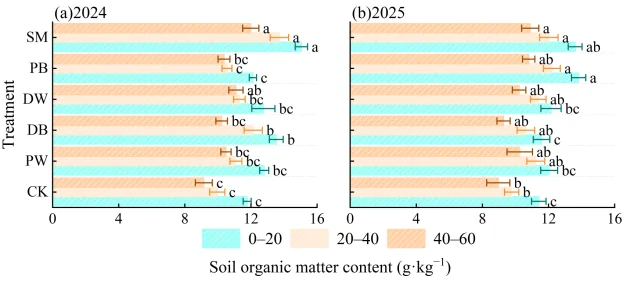
Soil organic matter content at different soil depths in the Indigowoad Root field under different mulching treatments. Values are means ± SE (n = 3). Different lowercase letters indicate significant differences among treatments within the same soil layer and year (*p* < 0.05).

As shown in Figure 5, soil organic matter content decreased with increasing soil depth. In the 0–20 cm soil layer, SM had the highest soil organic matter content, which was 24% higher than that under CK (*p* < 0.05). Soil organic matter content under DB was 18% higher than that under CK (*p* < 0.05), whereas PW, PB, and DW did not differ significantly from CK (*p* > 0.05). In the 20–40 cm soil layer, soil organic matter content followed the order SM > DB > DW > PW > PB > CK. PW, PB, and DW did not differ significantly from CK. In the 40–60 cm soil layer, SM again had the highest soil organic matter content, which was 26% higher than that under CK. Soil organic matter content under DB was 16% higher than that under CK. Across soil depths, SM consistently had the highest soil organic matter content.

#### 3.3.2. Soil Ammonium Nitrogen

As shown in Table 1, soil ammonium nitrogen content was highest in the 0–20 cm soil layer and decreased with increasing soil depth. In the 0–20 cm layer, DB had the highest soil ammonium nitrogen content, which was 25% higher than that under CK (*p* < 0.05). Soil ammonium nitrogen content was 7%, 6%, and 21% higher under DW, PW, and PB, respectively, than under CK. SM did not differ significantly from CK (*p* > 0.05). In the 20–40 cm soil layer, DB had the highest soil ammonium nitrogen content, whereas DW, PW, and PB did not differ significantly from one another. In the 40–60 cm soil layer, soil ammonium nitrogen content was 29% higher under DB than under CK. No significant differences were observed among DW, PW, PB, and SM.

#### 3.3.3. Soil Nitrate Nitrogen

As shown in Table 1, soil nitrate nitrogen content decreased with increasing soil depth and was highest in the 0–20 cm soil layer. In this layer, soil nitrate nitrogen content ranged from 68.61 to 81.91 mg kg^−1^. DB had the highest content, which was 17% higher than that under CK (*p* < 0.05). No significant differences were observed among PW, PB, and DW (*p* > 0.05), and SM did not differ significantly from CK. In the 20–40 cm soil layer, soil nitrate nitrogen content was significantly higher under DB and PB than under CK. No significant differences were observed among PW, DW, SM, and CK. In the 40–60 cm soil layer, DB had the highest soil nitrate nitrogen content, which was 15% higher than that under CK, followed by PB. No significant differences were observed among DB, PB, DW, and PW.

#### 3.3.4. Soil Available Phosphorus

As shown in Table 1, soil available phosphorus content decreased with increasing soil depth. In the 0–20 cm soil layer, DB had the highest soil available phosphorus content, which was 44% higher than that under CK (*p* < 0.05), followed by PB. PW and SM did not differ significantly from CK (*p* > 0.05). In the 20–40 cm soil layer, DB had the highest soil available phosphorus content, whereas SM had the lowest. In the 40–60 cm soil layer, soil available phosphorus content was 33% and 27% higher under DB and PB, respectively, than under CK. Contents under PW, DW, and SM were lower than those under DB and PB but remained higher than that under CK. Overall, soil available phosphorus content decreased with increasing soil depth and was concentrated mainly in the upper soil layer.

#### 3.3.5. Soil Available Potassium

As shown in Table 1, soil available potassium content varied among treatments and soil layers. It was highest in the 0–20 cm soil layer and decreased progressively with increasing soil depth. In the 0–20 cm layer, DB had the highest soil available potassium content, which was 11% higher than that under CK (*p* < 0.05). No significant differences were observed among PW, PB, and DW (*p* > 0.05). Soil available potassium contents under SM and CK were relatively low. In the 20–40 cm soil layer, soil available potassium content followed the order DB > PB > PW > DW > SM > CK. In the 40–60 cm soil layer, DB again had the highest soil available potassium content, which was 6% higher than that under CK, followed by PB.

### 3.4. Effects of Different Mulching Practices on the Growth, Dry Matter Accumulation, and Yield of Indigowoad Root

#### 3.4.1. Effects of Different Mulching Practices on Leaf Area Index

**Figure 6:**
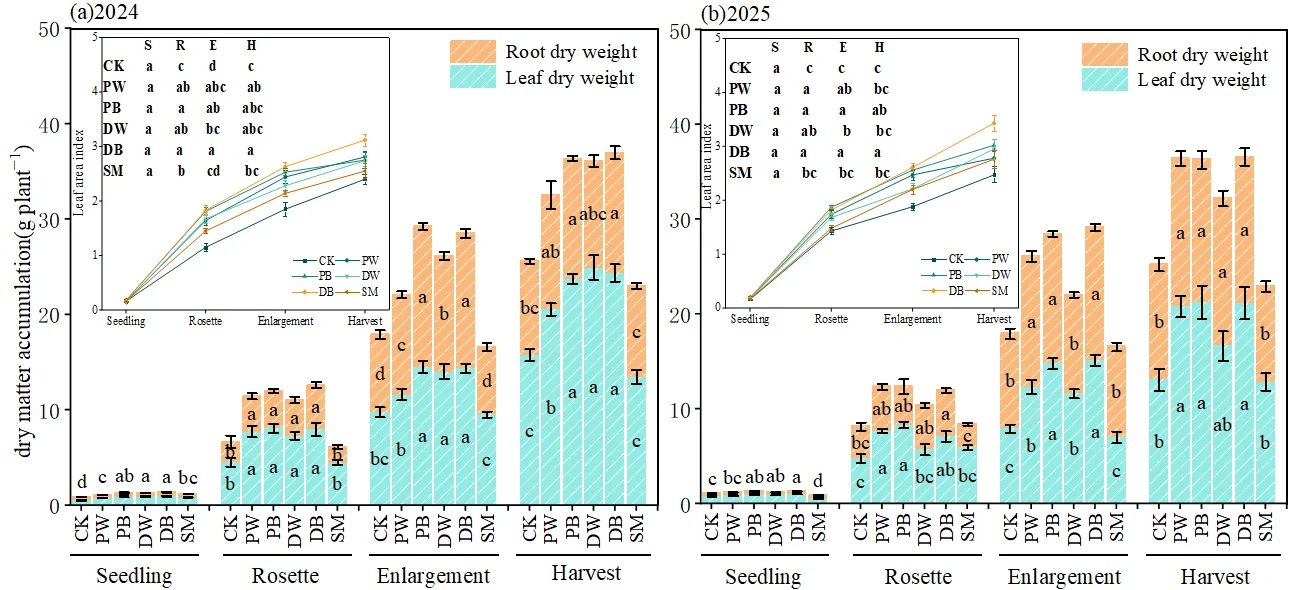
Effects of different mulching treatments on root and leaf dry matter accumulation and leaf area index at different growth stages of Indigowoad Root. Stacked bars show root and leaf dry matter accumulation, and the insets show leaf area index at different growth stages. Values are means ± SE (n = 3). Different lowercase letters indicate significant differences among treatments for the corresponding variable within the same growth stage and year (*p* < 0.05).

As shown in Figure 6, the leaf area index (LAI) of Indigowoad Root showed an increasing trend, with the treatments ranked as DB > PB > PW > DW > SM > CK. In most cases, LAI under all mulching treatments was higher than that under CK. LAI increased progressively from the seedling stage. At the rosette stage, DB, PB, DW, and PW significantly increased LAI by 43%, 44%, 31%, and 32% relative to CK, respectively (*p* < 0.05). No significant difference in LAI was observed between SM and CK (*p* > 0.05). At the fleshy-root expansion stage, DB and PB did not differ significantly from each other, and both had significantly higher LAI values than CK. At harvest, DB increased LAI by 34% relative to CK. Overall, DB was associated with greater leaf area development in Indigowoad Root.

#### 3.4.2. Effects of Different Mulching Practices on Dry Matter Accumulation in Indigowoad Root

Dry matter accumulation in Indigowoad Root is closely related to yield, and increasing dry matter accumulation is important for improving yield. Beginning at the rosette stage, dry matter accumulation increased progressively as plant growth advanced (Figure 6). At the rosette stage, dry matter accumulation under the black plastic film treatments, DB and PB, was significantly higher than that under CK (*p* < 0.05), with increases of 68% and 66%, respectively. Root dry matter accumulation under DB also differed significantly from that under CK and was 82% higher. In contrast, root dry matter accumulation under PW, PB, DW, and SM did not differ significantly from that under CK (*p* > 0.05). At the fleshy-root expansion stage, dry matter accumulation in Indigowoad Root increased further. Dry matter accumulation under the black plastic film treatments was greater than that under the white plastic film treatments. Among all treatments, dry matter accumulation was significantly higher under DB than under CK, with an increase of 61%, whereas SM did not differ significantly from CK. DB also significantly increased root dry matter accumulation by 57% relative to CK. At harvest, DB significantly increased dry matter accumulation by 45% relative to CK. These results indicate that biodegradable black plastic film mulching significantly increased dry matter accumulation in Indigowoad Root, whereas dry matter accumulation under straw mulching did not differ significantly from that under the bare-soil control.

#### 3.4.3. Effects of Different Mulching Practices on the Yield of Indigowoad Root

**Figure 7:**
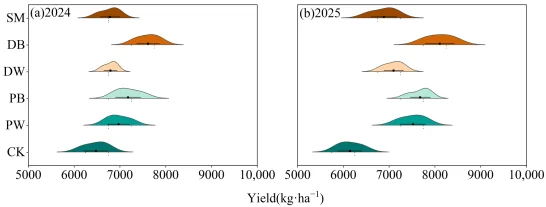
Distribution of the yield of Indigowoad Root under different mulching treatments. The density curves show the distributions of yield observations for each treatment, whereas the black points and horizontal lines summarize the center and spread of the corresponding distributions.

As shown in Figure 7, yield differed significantly among the mulching treatments. The black plastic film treatments, DB and PB, produced marked yield increases relative to the bare-soil control (CK). DB produced the highest yield, ranging from 7612.90 to 8106.06 kg ha^−1^, and significantly increased yield by 25% relative to CK (*p* < 0.05). Compared with CK, PB, PW, DW, and SM increased yield by 18%, 15%, 10%, and 8%, respectively. Yield under SM was similar to that under CK, with no significant difference between the two treatments (*p* > 0.05). These results show that yield responses varied among the different mulching practices, with DB showing an overall advantage in increasing the yield of Indigowoad Root.

### 3.5. Correlation and PLS-PM Analyses of Indigowoad Root Yield Formation

**Figure 8:**
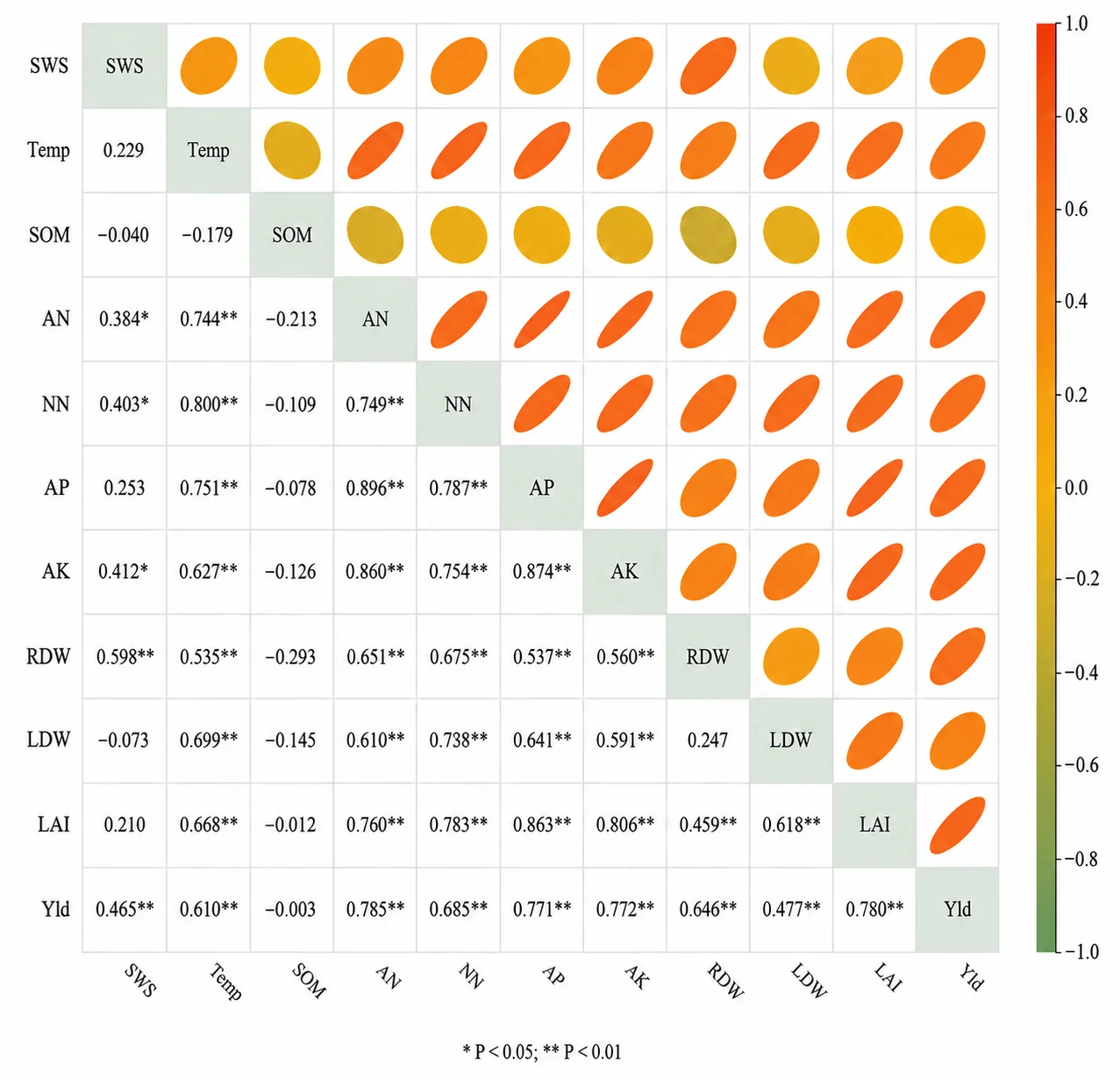
Correlations among soil environmental factors, soil nutrients, plant growth traits, and yield of Indigowoad Root under different mulching treatments. Numbers in the lower triangle are Pearson correlation coefficients. In the upper triangle, ellipse color indicates the direction of the correlation, with orange and green representing positive and negative correlations, respectively, and narrower ellipses indicating stronger correlations. SWS, soil water storage; Temp, soil temperature; SOM, soil organic matter; AN, ammonium nitrogen; NN, nitrate nitrogen; AP, available phosphorus; AK, available potassium; RDW, root dry weight; LDW, leaf dry weight; LAI, leaf area index; and Yld, fresh-root yield. * *p* < 0.05; ** *p* < 0.01.

**Figure 9:**
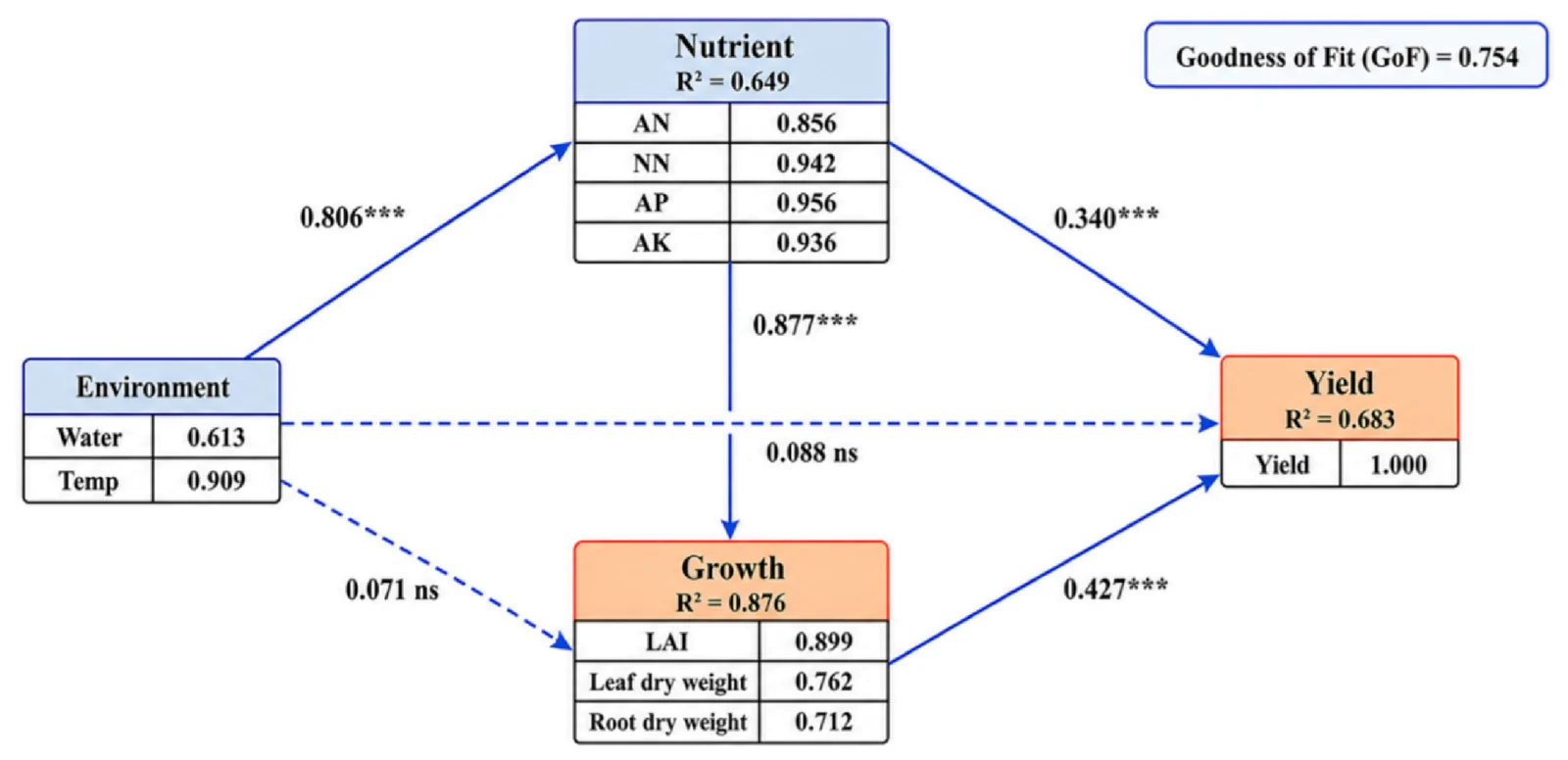
Partial least squares path model (PLS-PM) for Indigowoad Root under different mulching treatments. R^2^ indicates the proportion of variance in the dependent variable explained by the model. Solid blue arrows indicate significant positive paths, dashed blue arrows indicate nonsignificant positive paths, and “ns” indicates not significant (*p* > 0.05). Blue and orange boxes represent environmental/soil nutrient variables and plant growth/yield variables, respectively. *** indicates high significance at *p* < 0.001. GoF, goodness of fit.

Correlation analysis (Figure 8) showed that yield was positively correlated with ammonium nitrogen (AN), nitrate nitrogen (NN), available phosphorus (AP), available potassium (AK), root dry weight (RDW), leaf area index (LAI), leaf dry weight (LDW), soil temperature (Temp), and soil water storage (SWS) (*p* < 0.01), but was not significantly correlated with soil organic matter (SOM) (*p* > 0.05). The mineral nutrient indicators were also positively correlated with one another, with the strongest correlation occurring between AN and AP (r = 0.896), and were positively associated with Temp and, to a lesser extent, SWS. Assessing these intercorrelations was necessary because the environmental variables did not vary independently, and their individual correlations with yield should therefore not be interpreted as independent effects. RDW, LDW, and LAI were also positively correlated with Temp and the mineral nutrient indicators. The differences in the strength of the correlations of yield and RDW with the environmental parameters likely reflect their distinct biological meanings: yield is an integrative outcome of multiple growth components, whereas RDW specifically represents dry-matter accumulation in roots. Because pairwise correlations cannot distinguish direct from indirect associations, PLS-PM was further used to evaluate the potential pathways linking the soil environment, soil nutrients, plant growth, and yield (Figure 9). The model showed a good overall fit (GoF = 0.754). The soil environment was positively associated with soil nutrients (β = 0.806, *p* < 0.001), which were in turn positively associated with plant growth (β = 0.877, *p* < 0.001). Soil nutrients and plant growth were also positively associated with yield (β = 0.340 and 0.427, respectively; *p* < 0.001), whereas the direct paths from the soil environment to plant growth and yield were not significant (β = 0.071 and 0.088, respectively; *p* > 0.05). The model explained 64.9%, 87.6%, and 68.3% of the variance in soil nutrients, plant growth, and yield, respectively. The factor loadings ranged from 0.613 to 0.909 for the soil environment, from 0.856 to 0.956 for soil nutrients, and from 0.712 to 0.899 for plant growth. Overall, these results are consistent with an indirect pathway in which mulching-related variation in the soil hydrothermal environment was associated with Indigowoad Root yield through coordinated changes in soil nutrient status and plant growth.

## 4. Discussion

### 4.1. Effects of Different Mulching Practices on the Soil Hydrothermal Environment of Indigowoad Root

Soil water and temperature are major factors affecting crop growth and development. Plastic film mulching can reduce nonproductive soil water evaporation by limiting direct exchange between the soil and atmosphere, thereby conserving soil water [30,31]. Suitable soil hydrothermal conditions can support root growth and dry matter accumulation in Indigowoad Root and may consequently contribute to higher yield [31]. Zhang et al. [30] reported that black plastic film provided effective soil moisture conservation in the arid and semi-arid Hexi region, with soil water content under black film being significantly higher than that under white film. In the present study, soil water storage in the 0–60 cm soil profile was generally higher under DB than under the other treatments from the seedling stage to harvest. This result was consistent with the findings of Zhang et al. [30] and Pan et al. [32]. Compared with biodegradable white film, biodegradable black film generally has lower light transmittance and a weaker soil-warming effect. These properties may reduce soil water evaporation and improve soil moisture conservation [3,33]. DB may therefore help maintain water availability for the growth of Indigowoad Root [3,33].

Plastic film mulching can increase soil temperature by restricting moisture exchange across the film and modifying latent and sensible heat exchange. Water retained beneath the film may also attenuate longwave radiation from the soil at night and improve heat retention [34,35]. Indigowoad Root is a cool-season crop, and suitable soil temperatures are important for normal growth and fleshy-root formation. Excessively high or low temperatures may adversely affect its growth [34,36]. In this study, soil temperature decreased with increasing soil depth from the seedling stage to harvest. DW generally produced greater warming in the shallow soil layers than the other treatments, whereas DB limited the magnitude of shallow-soil warming relative to DW. This result was consistent with that of [33]. Air temperatures were comparatively high from the rosette stage to the fleshy-root expansion stage. Under these conditions, the relatively high shallow-soil temperature under DW might have accelerated phenological development and shortened the time available for fleshy-root expansion and dry matter accumulation, potentially increasing the risk of yield loss. Because phenological acceleration and heat injury were not measured directly, this explanation should be considered a possible mechanism rather than a demonstrated response.

DB provided moderate root-zone warming while limiting excessive warming of the shallow soil. This temperature pattern may have reduced the risk of heat stress and contributed to the higher yield under DB [33,34]. DW warmed the shallow soil more rapidly, whereas temperatures in deeper soil layers were generally similar under DW and DB. The comparatively large diurnal temperature range at Minle may partly explain why biodegradable black film could limit daytime warming while retaining heat at night. However, this interpretation requires confirmation using separate daytime and nighttime temperature data [33,35]. Soil temperatures under SM and CK were generally similar. Straw has low thermal conductivity and a loose, porous structure. It can reduce the amount of solar radiation reaching the soil surface, while air retained within the straw layer can slow heat transfer between the soil and atmosphere. These properties reduce the magnitude of daytime warming and buffer soil temperature fluctuations [37]. During the cool early growth period in the Hexi oasis region, however, straw mulching may delay soil warming and consequently provide less warming than plastic film mulching. Overall, DB combined relatively effective soil water conservation with moderate soil warming, conditions that may have supported Indigowoad Root growth and yield formation.

### 4.2. Effects of Different Mulching Practices on Soil Nutrients

Soil nutrient status is an important determinant of crop growth and development. Yang et al. [37] reported that straw and plastic film mulching can improve soil fertility by promoting soil organic matter accumulation and nutrient transformation. In the present study, the measured available nutrient contents after sowing were lower than their presowing levels in the soil layer for which baseline data were available. This decline occurred under both plastic film and straw-mulched conditions and should therefore be interpreted as a temporal change rather than as evidence that mulching caused nutrient depletion. Despite this general decline, DB maintained relatively high ammonium nitrogen, nitrate nitrogen, available phosphorus, and available potassium contents compared with CK, particularly in the 0–20 cm soil layer. SM had the highest soil organic matter content. These findings suggest that the effects of mulching differed between soil organic matter accumulation and available nutrient status, with the clearest treatment differences occurring in the upper soil layer. The relatively high soil organic matter content under SM may be associated with the direct input and microbial decomposition of straw. Straw decomposition can add organic matter to the soil, modify soil structure, and contribute to nutrient supply [36,38]. Because microbial biomass and decomposition rates were not measured, these processes should be considered literature-supported explanations for the observed soil organic matter pattern. DB was associated with relatively high available nutrient contents in shallow soil. One possible explanation is that the soil water and temperature conditions under DB were favorable for microbial activity, enzymatic reactions, organic matter mineralization, and the dissolution and release of soil nutrients [32,33,37]. These processes were not measured directly in the present study and therefore remain plausible mechanisms rather than demonstrated causal pathways.

Overall, SM had a clear advantage in increasing soil organic matter content, suggesting potential benefits for the soil organic carbon pool, although soil organic carbon stocks were not measured directly. DB, by contrast, was associated with relatively high available nutrient contents. The hydrothermal and nutrient conditions under DB may have supported root growth, leaf area expansion, and dry matter accumulation, thereby contributing to yield formation.

### 4.3. Effects of Different Mulching Practices on Canopy Development, Dry Matter Accumulation, and Yield of Indigowoad Root

Dry matter accumulated in Indigowoad Root is derived from photosynthetic assimilates and depends largely on canopy characteristics [39]. Leaf area is an important factor affecting dry matter accumulation and yield formation. In the present study, LAI was generally higher under the mulching treatments than under CK, although the magnitude and statistical significance of the differences varied among treatments and growth stages. LAI under DB was significantly higher than that under CK at the rosette and fleshy-root expansion stages, consistent with the findings of Qin et al. [40]. The relatively high LAI under DB may be associated with its combined effects on soil water and temperature. Black plastic film can help maintain plow-layer soil temperature within a physiologically favorable range while conserving soil water [33]. DB may therefore alleviate constraints imposed by low temperatures during early growth, reduce soil water evaporation, and maintain root-zone water availability [30,33]. Together, these effects could promote leaf expansion and canopy development and thereby support dry matter accumulation. Plastic film mulching is generally considered beneficial for dry matter accumulation because of its soil-warming and moisture-conservation effects. Final dry matter mass depends on both the rate and duration of dry matter accumulation [33]. In the semi-arid study region, however, conditions were cool during early growth. Although straw mulching conserved soil moisture, it also delayed the warming of the surface soil [37]. This cooling effect might reduce root activity and nutrient uptake and delay leaf area expansion and photosynthetic assimilate production, thereby restricting dry matter accumulation and yield formation [41]. Root activity, nutrient uptake, and photosynthetic assimilate production were not measured directly; these explanations should therefore be interpreted cautiously.

The PLS-PM characterized the relationships among soil environment, soil nutrients, plant growth, and yield. The soil environment had a significant positive path to soil nutrients (β = 0.806, *p* < 0.001), whereas its direct paths to plant growth and yield were not significant. This pattern was consistent with relationships in which the soil environment was linked to plant growth and yield through soil nutrient status, although confirmation of mediation would require the corresponding indirect effects and bootstrap confidence intervals [32]. DB produced the highest yield. This response may partly reflect the temperature-buffering effect of biodegradable black film, which may have improved root-zone hydrothermal conditions and facilitated soil nutrient transformation and root nutrient uptake. These changes could have provided favorable nutrient conditions for canopy development and dry matter accumulation [32,33]. Soil nutrients had a strong positive path to the plant growth construct (β = 0.877, *p* < 0.001) and a direct positive path to yield (β = 0.340, *p* < 0.001). Plant growth, represented by LAI, leaf dry weight, and root dry weight, also had a positive path to yield (β = 0.427, *p* < 0.001). LAI had the highest loading within the plant growth construct, indicating that it was an important indicator of this construct. Greater leaf area can enhance light interception and photosynthetic assimilate production, thereby supporting dry matter accumulation and yield formation [39].

The higher LAI, dry matter accumulation, and yield observed under DB were consistent with the relationships represented by the PLS-PM. Collectively, the results suggest that DB-related changes in soil hydrothermal conditions and nutrient status may have supported canopy development and dry matter accumulation, which in turn may have contributed to higher yield. Because mulching treatment was not directly included mulching treatment as a construct, it should not be interpreted as proving a causal mechanism specific to DB.

It should be noted that this study was conducted using Indigowoad Root in Minle, Gansu Province, and therefore provides a theoretical basis for understanding the effects of mulching under similar climatic conditions. For practical application, the specific technical parameters of this mulching strategy should be locally validated and adjusted according to climatic conditions during the crop-growing season. Moreover, the degradation rate, residual amount, and long-term soil effects of biodegradable films were not monitored. Future studies will incorporate film degradation and residue assessments into the same experimental framework to further clarify differences in the yield-enhancing effects of various film-mulching strategies and their underlying mechanisms.

## 5. Conclusions

(1) Biodegradable black plastic film mulching (DB) provided effective soil moisture conservation. Across the growth stages, soil water storage was generally higher under DB than under the other treatments. Biodegradable white plastic film mulching (DW) generally produced the greatest warming in the shallow soil layers, whereas the treatment producing the highest temperature in deeper layers varied with growth stage and soil depth.
(2) DB was associated with relatively high available nutrient contents, particularly in the 0–20 cm soil layer, and supported canopy development, dry matter accumulation, and yield formation in Indigowoad Root. SM had an advantage in increasing soil organic matter content, but dry matter accumulation and yield under SM did not differ significantly from those under CK. Yield was 17.5–31.9% higher under DB than under CK.
(3) The PLS-PM supported relationships linking the soil hydrothermal environment to nutrient status, plant growth, and yield. Soil nutrients were linked to yield both directly and through plant growth. These paths should be interpreted as model-based relationships rather than proof of a causal mechanism.
(4) Among the biodegradable-film treatments, DB showed more favorable overall performance than DW under the conditions of this two-year field experiment. DB and DW may be considered candidate practices for Indigowoad Root production in the Zhangye region, but broader adoption requires validation across additional sites and years.

## Figures and Tables

**Table 1:** Soil ammonium nitrogen, nitrate nitrogen, available phosphorus, and available potassium contents at different soil depths in the Indigowoad Root field under different mulching treatments. CK, bare soil without mulching (control); PW, white polyethylene film mulching; PB, black polyethylene film mulching; DW, biodegradable white plastic film mulching; DB, biodegradable black plastic film mulching; SM, straw mulching; AN, ammonium nitrogen; NN, nitrate nitrogen; AP, available phosphorus; and AK, available potassium.Different lowercase letters indicate significant differences among treatments for the same nutrient within the same year and soil layer (*p* < 0.05).

Year	Treatment	AN (mg kg^−1^)			NN (mg kg^−1^)			AP (mg kg^−1^)			AK (mg kg^−1^)		
Soil Depth (cm)		0–20	20–40	40–60	0–20	20–40	40–60	0–20	20–40	40–60	0–20	20–40	40–60
2024	PW	54.2 ± 2.9 b	51.8 ± 3.1 b	45.6 ± 2.4 b	77.4 ± 3.9 b	60.7 ± 2.9 bc	52.4 ± 2.7 b	32.7 ± 1.8 d	18.6 ± 1.4 c	17.8 ± 1.5 bc	103.7 ± 5.4 cd	91.8 ± 4.2 bc	83.8 ± 3.9 ab
	PB	62.0 ± 2.9 a	54.1 ± 2.3 b	48.8 ± 2.3 b	78.9 ± 3.8 ab	65.3 ± 2.7 a	53.8 ± 2.8 ab	39.7 ± 1.8 b	21.7 ± 1.8 ab	19.6 ± 1.7 ab	108.0 ± 5.8 b	92.9 ± 4.4 ab	85.0 ± 3.9 a
	DW	54.4 ± 2.8 b	51.6 ± 3.0 b	47.6 ± 2.7 b	79.7 ± 3.7 ab	61.8 ± 2.8 b	52.7 ± 1.9 b	36.9 ± 1.7 c	19.7 ± 1.7 bc	16.0 ± 1.2 cd	105.7 ± 6.2 bc	90.6 ± 3.8 bc	81.4 ± 4.2 bc
	DB	63.6 ± 2.8 a	59.1 ± 3.0 a	52.6 ± 3.3 a	80.7 ± 3.9 a	66.7 ± 2.8 a	55.4 ± 2.8 a	43.2 ± 2.0 a	22.2 ± 0.7 a	20.4 ± 0.7 a	111.6 ± 5.7 a	95.1 ± 4.8 a	84.9 ± 3.8 a
	SM	49.4 ± 3.1 c	47.0 ± 2.4 c	41.7 ± 2.7 c	69.6 ± 2.8 c	58.5 ± 2.6 bc	49.4 ± 1.8 c	32.5 ± 1.5 d	15.1 ± 0.8 d	15.7 ± 0.8 d	102.6 ± 5.8 d	90.5 ± 4.8 bc	79.4 ± 4.4 c
	CK	50.8 ± 2.8 c	45.7 ± 2.8 c	41.4 ± 2.2 c	68.6 ± 2.8 c	57.5 ± 2.4 c	47.6 ± 2.2 c	29.4 ± 1.2 d	17.6 ± 0.7 c	15.3 ± 0.6 d	99.9 ± 5.1 e	89.7 ± 4.9 c	80.7 ± 4.5 c
2025	PW	57.0 ± 3.0 bc	55.4 ± 2.2 b	47.9 ± 2.0 bc	79.4 ± 3.2 a	61.9 ± 2.1 bc	53.2 ± 2.4 ab	33.5 ± 1.1 c	19.2 ± 1.2 ab	18.0 ± 0.7 ab	106.8 ± 5.2 bc	93.8 ± 4.5 ab	85.1 ± 4.1 ab
	PB	64.9 ± 3.0 a	57.5 ± 2.3 b	51.4 ± 2.2 b	80.5 ± 3.6 a	67.8 ± 2.9 a	54.5 ± 2.7 ab	41.4 ± 1.9 ab	22.1 ± 1.7 a	19.8 ± 1.5 a	109.5 ± 5.4 b	94.4 ± 4.5 ab	86.0 ± 4.7 a
	DW	57.6 ± 2.8 b	54.7 ± 2.0 b	51.8 ± 2.5 b	81.0 ± 4.0 a	63.2 ± 3.2 b	53.6 ± 2.5 ab	38.0 ± 1.7 b	20.2 ± 1.1 ab	16.1 ± 1.0 b	107.3 ± 5.1 bc	93.0 ± 4.2 bc	82.6 ± 4.5 b
	DB	67.4 ± 3.1 a	62.4 ± 2.0 a	57.7 ± 2.1 a	81.9 ± 3.4 a	68.4 ± 3.2 a	55.4 ± 2.2 a	43.4 ± 1.8 a	22.3 ± 1.4 a	20.5 ± 0.8 a	113.2 ± 5.0 a	96.5 ± 4.1 a	87.9 ± 4.4 a
	SM	53.3 ± 2.2 d	49.4 ± 2.5 c	48.7 ± 1.9 b	71.5 ± 3.2 b	60.1 ± 2.3 bc	50.9 ± 1.9 bc	32.4 ± 1.9 c	15.5 ± 1.2 b	16.1 ± 0.9 b	104.4 ± 5.8 cd	93.4 ± 4.7 bc	76.8 ± 3.7 c
	CK	54.4 ± 2.9 cd	49.2 ± 2.8 c	44.1 ± 2.4 c	70.2 ± 3.9 b	58.9 ± 2.5 c	49.1 ± 1.7 c	30.7 ± 1.2 c	18.5 ± 1.4 ab	15.6 ± 1.2 b	102.7 ± 5.2 d	91.4 ± 4.7 c	82.2 ± 3.5 b

## Data Availability

The original contributions presented in this study are included in the article. Further inquiries can be directed to the corresponding authors.
